# Invasive crayfish impacts on native fish diet and growth vary with fish life stage

**DOI:** 10.1007/s00027-016-0483-2

**Published:** 2016-04-22

**Authors:** Kevin A. Wood, Richard B. Hayes, Judy England, Jonathan Grey

**Affiliations:** 1 School of Biological and Chemical Sciences, Queen Mary University of London, London, E1 4NS UK; 2 Wildfowl and Wetlands Trust, Slimbridge, Slimbridge, Gloucestershire GL2 7BT UK; 3 Environment Agency, Apollo Court, 2 Bishops Square, St Albans Road West, Hatfield, AL10 9EX UK; 4 The Wild Trout Trust, PO Box 120, Waterlooville, PO8 0WZ UK; 5 Lancaster Environment Centre, Lancaster University, Lancaster, LA1 4YQ UK

**Keywords:** Chub *Squalius cephalus*, Competition, Diet shift, Invasive species, Scalimetry, Signal crayfish *Pacifastacus leniusculus*, Stable isotopes

## Abstract

Assessing the impacts of invasive organisms is a major challenge in ecology. Some widespread invasive species such as crayfish are potential competitors and reciprocal predators of ecologically and recreationally important native fish species. Here, we examine the effects of signal crayfish (*Pacifastacus leniusculus*) on the growth, diet, and trophic position of the chub (*Squalius cephalus*) in four rivers in Britain. Growth rates of 0+ chub were typically lower in sympatric populations with signal crayfish compared with allopatric populations, and this effect could be traced through to 2+ chub in one river. However, growth rates of older chub (5+ to 6+) were typically higher in the presence of crayfish. Sympatry with crayfish resulted in lower chub length-at-age and mass-at-age in half of the rivers sampled, with no change detected in the other rivers. Stable isotope analyses (δ^13^C and δ^15^N) revealed that both chub and crayfish were omnivorous, feeding at multiple trophic levels and occupying similar trophic positions. We found some evidence that chub trophic position was greater at invaded sites on one river, with no difference detected on a second river. Mixing models suggested crayfish were important food items for both small and large chub at invaded sites. This study provides evidence that invasive species can have both positive and negative effects on different life stages of a native species, with the net impact likely to depend on responses at the population level.

**Electronic supplementary material:**

The online version of this article (doi:10.1007/s00027-016-0483-2) contains supplementary material, which is available to authorized users.

## Introduction

The spread of organisms beyond their natural geographic range is a serious global threat causing both ecological and economic damage (Clavero and Garcia-Berthou 2005; Roy et al. 2012) and rates of invasion show little sign of abating in some systems (e.g. Jackson and Grey 2013). Aquatic ecosystems are particularly vulnerable to the impacts of invasive organisms; the spread of non-native species is often facilitated by human activities and by the rapid dispersal possible in water (Rahel 2007; Strayer and Dudgeon 2010). A number of recent studies have demonstrated that invasive species may increase or decrease the growth rates, and alter the diets of, native organisms through several key mechanisms, including competition, predation, and transmission of pathogens (e.g. Corrreia 2001; King et al. 2006; Maguire and Grey 2006). Where prey availability is affected, a dietary shift to a different or previously under exploited prey resource may occur in order to maintain foraging efficiency (Syväranta and Jones 2008).

Owing to their omnivory, large body size and potential to dominate benthic biomass, some crayfish have become key invasive species that can affect ecosystem processes, services, and biodiversity, as well as the abundance, distribution, growth, diet and behaviour of native organisms (Lodge et al. 2000). North American signal crayfish (*Pacifastacus leniusculus* D. 1852) were introduced into Europe in the 1970s for aquaculture and represent one of the most widespread non-indigenous crayfish species (Holdich et al. 2009). Research has tended to focus on the interactions between signal and native crayfish (Holdich et al. 2009; Olsson et al. 2009a; Ercoli et al. 2014) and more recently with other invasive crayfish (Jackson et al. 2014); consequently less is known about interactions with fishes. In rivers, signal crayfish may reduce the growth and abundance of small benthic fishes, through interspecific competition and predation (Guan and Wiles 1997; Light 2005), and out-compete fish for refugia (Griffiths et al. 2004); yet other research has reported no effects of invasive crayfish on juvenile fish survival (Stenroth and Nyström 2003). However, there have been relatively few attempts to assess the specific impacts that signal crayfish may have on larger fish species of ecological and recreational importance (Reynolds 2011; Ruokonen et al. 2012; but see Bašić et al. 2015). Understanding the full-range of crayfish-fish interactions, and both the individual- and population-level consequences are essential if fish populations are to be managed successfully.

Our study compared the growth, diet, and trophic position of a native predatory fish when found in allopatry and sympatry with signal crayfish, and tested three hypotheses. Our first hypothesis was that fish growth rates would be lower at invaded sites because signal crayfish have been shown to reduce the availability of many prey taxa, such as aquatic invertebrates (Stenroth and Nyström 2003; Crawford et al. 2006), benthic fishes (Guan and Wiles 1997), and macrophytes (Nyström et al. 1996). Such changes in prey availability led to our second hypothesis; that fish diet would change after crayfish invasion by shifting to increased use of prey items typically unavailable to crayfish, such as terrestrial invertebrates. Our third hypothesis was that the impacts of crayfish upon fish would be greater for smaller relative to larger individuals via reciprocal predation as well as competition, with each species consuming particular life stages of the other species. Crayfish predominantly feed on fish eggs and larvae, but will also attack small individuals, whereas fish consumption of crayfish typically increases with fork length and hence is greater for adult fish (Hellawell 1971b; Blake and Hart 1995; García-Berthou 2002; Gladman et al. 2012).

We tested our hypotheses using chub (*Squalius cephalus* L.), native to rivers across Europe, and a potential competitor and reciprocal predator of invasive crayfish. Chub are omnivorous, foraging on aquatic and terrestrial invertebrates, macrophytes, detritus, fishes and other small vertebrates (Hellawell 1971b; Mann 1976) and are popular with anglers. Impacts on chub growth and feeding could alter food web structure, energy flow, community composition and the recreational value of lowland rivers. Thus, juvenile chub would experience reduced prey availability and increased predation pressure, whereas larger chub would experience smaller reductions in prey availability (with larger gape increasing prey range) and this would be partially offset by the greater inclusion of signal crayfish in their diet (Nyström et al. 2006).

## Materials and methods

We used two complementary study approaches to assess the effects of signal crayfish on the growth, condition, and trophic position of chub in four lowland British rivers (Table 1). For two rivers (Evenlode and Cherwell), we used a before–after approach to compare effects on chub before and after signal crayfish invasion. From a further two rivers (Rother and Chad Brook), we used a space-for-time approach in which chub from sites with established signal crayfish populations were compared with chub from uninvaded sites upstream on those rivers; within each river we selected invaded and uninvaded sites with comparable hydrological conditions (i.e. discharge), physical structure (i.e. channel width, depth), land use, and ecological communities, in order to avoid such differences confounding our ability to detect the effects of crayfish on chub. Signal crayfish were first recorded in 2000 and 1995, in the Evenlode and Cherwell, respectively, and thus archived scales provided by the Environment Agency from chub caught before 2000 (Evenlode) and 1995 (Cherwell) were used to obtain pre-invasion growth data, while scales from chub spawned after 2000 and 1995 were used to obtain post-invasion data (Environment Agency data 2008). The Rother was invaded by signal crayfish between 1973 and 1975 (Environment Agency data 2008). Extensive sampling indicated that the invaded stretch extended from a weir (51°00′15.16″N, 00°53′04.96″W) downstream to 51°00′15.07″N, 00°52′54.70″W; immediately upstream of the weir, from 51°00′11.93″N, 00°53′05.04″W to 51°00′09.03″N, 00°53′41.02″W was uninvaded. Signal crayfish invaded Chad Brook from the confluence with the River Stour after 2000 to a weir at 52°04′43.71″N, 00°42′54.31″E (Environment Agency data 2008). Thus, the river above the weir to 52°04′49.33″N, 00°43′31.43″E was designated as the uninvaded site, while the river below the weir to 52°26′10.99″N, 00°43′46.80″E was classified as the invaded site.

**Table 1:** A summary of key characteristics associated with each of our four study rivers (Environment Agency data 2008)

Parameter	Evenlode	Cherwell	Chad Brook	Rother
Catchment area (km^2^)	430.0	943.0	47.4	346.0
Length (km)	39.5	64.4	14.4	52.0
Mean annual discharge (m^3^ s^−1^)	3.8	5.5	0.3	2.3
Dominant land use	Arable and pastoral agriculture	Arable and pastoral agriculture	Arable agriculture	Arable and pastoral agriculture
Year crayfish invasion first detected	2000	1995	2000	1975
Study approach used	Before-after	Before-after	Space-for-time	Space-for-time
Scalimetry used?	Yes	Yes	Yes	Yes
Stable isotope analysis used?	No	No	Yes	Yes
No. chub (non-invaded site)	28	24	21	14
No. chub (invaded site)	40	34	15	18

### Growth rates

Age estimation based on annuli counts from calcified tissues such as scales has been routinely used for chub (Hellawell 1971a; Mann 1976). Scale-derived growth data allow long-term assessment of the effects of perturbations (i.e. growth pre- and post-crayfish invasion). Chub were sampled by angling in the Rother (n = 32) and Chad Brook (n = 36) during June–September in 2 years: 2008 and 2011. Mass (±1 g) and fork length (±1 mm) were determined in the field and three scales were removed from each chub from the flank between the dorsal fin and lateral line. All individuals were returned alive. For the Rivers Evenlode (n = 68) and Cherwell (n = 58), archived scales provided by the Environment Agency from chub caught before 2000 (Evenlode) and 1995 (Cherwell) were used to obtain pre-invasion growth data, while scales from chub spawned after 2000 and 1995 were used to obtain post-invasion data. Scales were examined using a SMZ1000 dissection microscope (Nikon, Japan) and estimates of length-at-age were back calculated using the Fraser-Lee formula, assuming a length of first scale formation of 15.9 mm (Economou et al. 1991).

### Stable isotope analyses

Stable isotope ratios of carbon and nitrogen vary in a conservative, predictable manner between trophic levels and thus changes in those ratios can be an effective technique in assessing dietary shifts of consumers in response to the invasion of an ecosystem by an alien species (Jackson et al. 2012). Non-destructive sampling is facilitated where tissue such as scales can be sampled, making stable isotope analysis an ideal investigative tool for aquatic ecosystems with small fish populations of conservational or recreational value (Perga and Gerdeaux 2003; Grey et al. 2009). We used the baseline-corrected estimates of trophic height (sensu Cohen et al. 2003) to compare the trophic position of chub between sites with and without invasive crayfish and mixing models to determine relative contributions from food sources. We combined these complementary methods, assessing growth rates by traditional techniques of scalimetry and then analysing the recent (<2 years) material for stable isotopes sequestered in the scales (Grey et al. 2009). Scale isotope ratios were converted to muscle ratios to facilitate the comparison with crayfish and prey species.

We analysed δ^13^C and δ^15^N of chub scales, crayfish, and putative prey to assess the trophic position of chub and crayfish, their diets, and potential dietary overlap. Qualitative sampling was carried out in May 2008 and June 2011 at invaded and uninvaded sites on the Rother and Chad Brook to collect potential dietary resources. Aquatic invertebrates (min. *n* = 5 individuals pooled per taxa), macrophytes (*n* = 5 leaves pooled from different individual plants of the dominant species present), and small fish (*n* ≥ 5 per species) were obtained by kick sampling; terrestrial invertebrates (*n* = 5 individuals pooled per species) were obtained by sweeping riparian vegetation with a butterfly net. Detritus (~250 g) was taken from the main channel substrate. Signal crayfish were also collected from invaded sites at Chad Brook (*n* = 18) and the Rother (*n* = 19) by kick-sampling. Carapace length was determined for each individual by measuring from the rostrum tip to carapace posterior. All samples except chub scales were frozen at −20 °C until preparation for stable isotope analysis. A portion of the outer section of each scale, equivalent to the most recent two annuli, was removed for stable isotope analysis. Each sample was macerated in a glass vial and oven dried at 60 °C for 48 h, then pulverised using an agate mortar and pestle, and 0.6 ± 0.05 mg weighed into tin cups. Samples were combusted using an elemental analyser (Flash EA, 1112 series, Thermo-Finnigan) coupled to a continuous flow isotope ratio mass spectrometer (Finnigan MAT DeltaPlus, Thermo-Finnigan).

Chub and crayfish stable isotope ratios were derived from scale and muscle, respectively. Both δ^13^C and δ^15^N vary between tissue types, but previous studies have shown that there is a dependable relationship between fish muscle and scale (e.g. Grey et al. 2009). Therefore, to better compare chub to their diet and to the crayfish, a conversion factor was derived from the stable isotope ratios for both scale and muscle tissue. Fifteen chub of three age classes (0+, 1+, and 2+; *n* = 5 for each class) from Calverton Fish Farm (Nottingham, UK), were sacrificed; muscle was excised from the left flank above the lateral line, and both scale and muscle samples prepared as above.

### Statistical analyses, isotope-metrics and mixing models

Statistical analyses were performed using R version 3.1.2 (R Development Core Team 2015), with significant effects attributed where *p* < 0.05. For both our before–after invasion sites (Evenlode and Cherwell) and our space-for-time sites (Rother and Chad Brook) we tested the effects of site (invaded versus uninvaded) and sampling year (2008 vs 2011; Rother and Chad Brook only) on (1) chub growth rates for each age-class, and (2) trophic position (baseline-corrected δ^15^N), using linear models with Gaussian error structures. Site and year were treated as fixed factors. Analysis of covariance (ANCOVA) was used to test for differences in the relationships between (1) fork length and age, (2) mass and age, and (3) trophic position and fork length, between invaded and uninvaded sites. Sampling year (2008 or 2011) was also included as a covariate. Normality and equality of variances were ascertained for residuals via Anderson–Darling and Levene’s tests, respectively. Linear regressions were plotted through six basal consumers (invertebrates; three terrestrial, three aquatic) for both the Rother (Aquatic: Trichoptera, Amphipoda, Ephemeroptera; Terrestrial: Coleoptera, Hemiptera, Hymenoptera) and Chad Brook (Aquatic: Gastropoda, Amphipoda, Heteroptera; Terrestrial: Coleoptera, Hemiptera, Diptera), and the perpendicular distance from the generated sloping baseline to the chub or crayfish (measured as change along the δ^15^N axis) gave the trophic height for each individual.

Chub diet shifts ontogenetically, with Hellawell (1971b) reporting that ≤5+ chub consumed greater proportions of terrestrial and aquatic invertebrates (excluding crayfish), and less plant matter than ≥6+ chub. The mean length of a 5+ chub, based on data from this study and a meta-analysis of chub length-at-age data (Mann 1976) was 231.7 mm. Thus, to account for potential ontogenetic shifts, chub were classified on fork length as either small (<232 mm; Rother, uninvaded *n* = 4, invaded *n* = 5; Chad Brook, uninvaded *n* = 2, invaded *n* = 12) or large (≥232 mm; Rother, uninvaded *n* = 10, invaded *n* = 13; Chad Brook, uninvaded *n* = 19, invaded *n* = 3). The baseline regressions described earlier were used to estimate the mean (±95 % CI) trophic height of chub and crayfish populations.

SIAR mixing model fractionation values (Parnell et al. 2010) were derived as follows. A mean Δ^13^C value (2.1 ‰) was calculated from four controlled feeding studies (*Coregonus nasus*: +2.0 ‰, Hesslein et al. 1993; *Oncorhynchus mykiss*: +1.3 ‰, Rounick and Hicks 1985; *Oncorhynchus mykiss*: +1.9 ‰; *Salvelinus fontinalis*: +3.3 ‰ McCutchan Jr et al. 2003). As chub are omnivorous and the fractionation can be dependent on the nitrogen content of food items, a value of +2.3 ‰ was used for Δ^15^N following McCutchan Jr et al. (2003). These values were added to all source items under the following categories: crayfish, macrophytes, detritus, terrestrial invertebrates, small fish, and aquatic invertebrates. Cannibalism is not thought to be common among chub (Hellawell 1971b; Mann 1976), and as few individuals were large enough to ingest any other within our samples, cannibalism was excluded from the analysis.

Although crayfish diet has also been reported to vary ontogenetically (Guan and Wiles 1997; Bondar et al. 2005), analysing crayfish in various size classes had negligible effects on SIAR output and therefore crayfish were analysed as a single group. A Δ^13^C value of +2.0 ‰ was taken from a feeding experiment using *Procambarus clarkii* (Rudnick and Resh 2005) while +2.3 ‰ was used once more for nitrogen for the same reasons as for chub. As cannibalism in signal crayfish has been reported, crayfish were included as a potential food source (Guan and Wiles 1997; Stenroth and Nyström 2003).

## Results

**Fig. 1:**
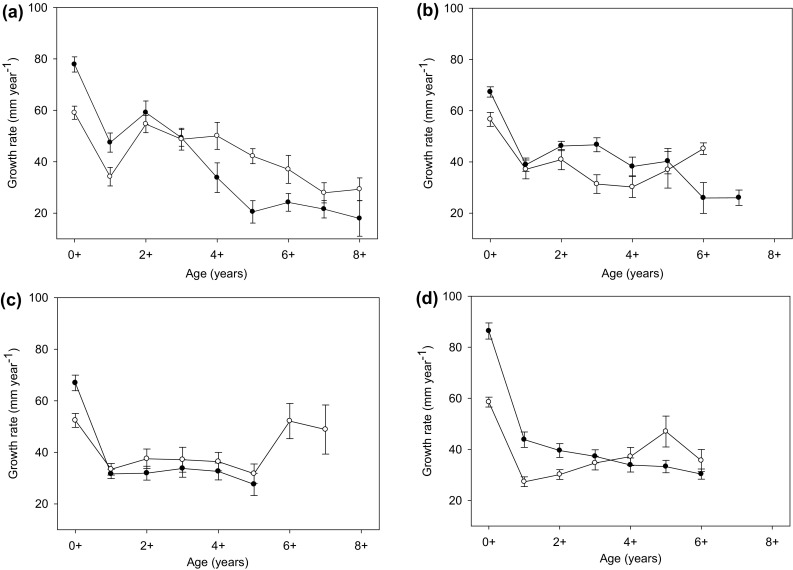
A comparison of calculated mean (±SE) yearly growth rates of chub sampled from uninvaded (*closed symbols*) and invaded (*open symbols*) sites on **a** the Rother, **b** Chad Brook, **c** the Cherwell, and **d** the Evenlode

**Fig. 2:**
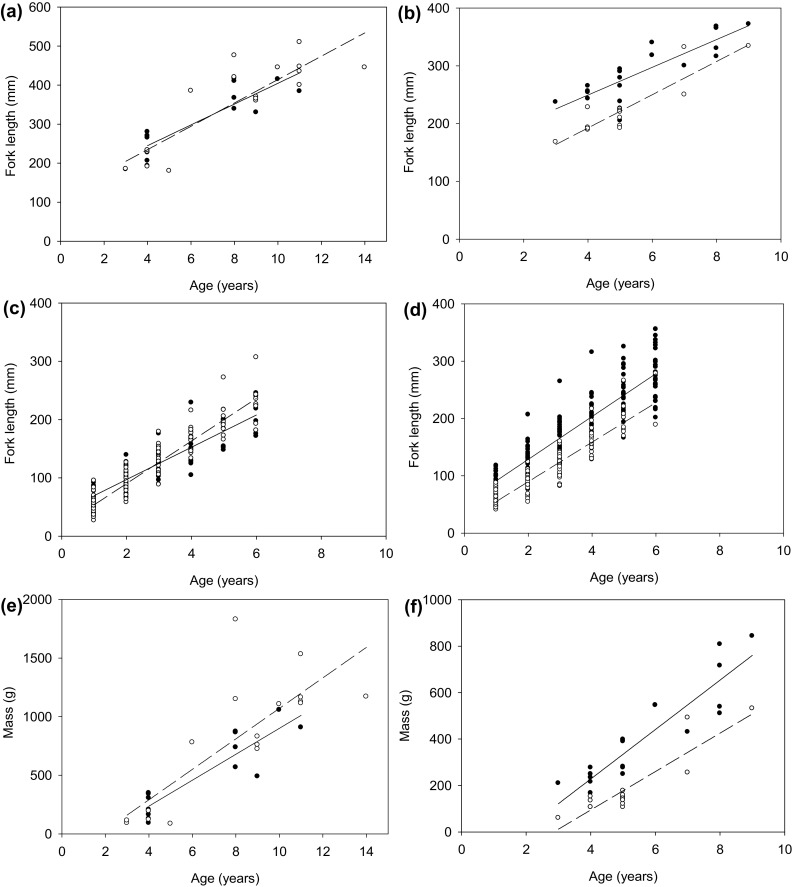
Chub fork length-at-age at uninvaded (*solid circles* and *line*) and invaded (*open circles* and *dashed line*) sites on **a** the Rother, **b** Chad Brook, **c** the Cherwell, and **d** the Evenlode. Chub mass-at-age at uninvaded (*solid circles* and *line*) and invaded (*open circles* and *dashed line*) sites on **e** the Rother and **f** Chad Brook

Chub from uninvaded sites on all four rivers exhibited decreasing annual growth rate with increasing age (Fig. 1). However, this pattern did not hold for the invaded sections on three of our four rivers, where annual growth rates increased again at ages of 5+ or above (site dependent). Chub growth rates were significantly lower at invaded relative to uninvaded sites for 0+ chub in all rivers (Table 2). Whilst there were indications of lower growth rates in 1+ and 2+ chub in some invaded river sites, growth rates were only significantly lower in the Evenlode. Older fish from invaded sites exhibited accelerated growth rates compared to uninvaded sites in the Rother at ages 5+ and 6+, at 6+ in Chad Brook, and at 5+ in the Evenlode (Table 2). Chub growth rates differed significantly between 2008- and 2011-sampled fish in only 3 out of 13 models; 1+ Rother, 3+ and 4+ Chad Brook. Significantly greater fork length-at-age at uninvaded relative to invaded sites was found for Chad Brook and Evenlode (Table 3; Fig. 2). Furthermore, Chad Brook chub achieved greater mass-at-age at uninvaded relative to invaded sites (Table 3; Fig. 2).

**Table 2:** The effects of site (invaded versus uninvaded) and year (2008 vs 2011) on chub growth rates, as indicated by linear models

Age class	Factor	Rother	Chad Brook	Cherwell	Evenlode
0+	Site	***F***_**1,30**_ = **11.44**; ***p*** = **0.002**	***F***_**1,34**_ = **13.94**; ***p*** = **0.001**	***F***_**1,56**_ = **12.63**; ***p*** = **0.001**	***F***_**1,66**_ = **62.95**; ***p*** < **0.001**
	Year	*F*_1,30_ = 2.84; *p* = 0.103	*F*_1,34_ = 2.95; *p* = 0.095	–	–
1+	Site	*F*_1,30_ = 1.63; *p* = 0.212	*F*_1,34_ = 1.27; *p* = 0.268	*F*_1,49_ = 0.36; *p* = 0.552	***F***_**1,64**_ = **23.43**; ***p*** < **0.001**
	Year	***F***_**1,30**_ = **5.98**; ***p*** = **0.021**	*F*_1,34_ = 2.14; *p* = 0.153	–	–
2+	Site	*F*_1,30_ = 1.49; *p* = 0.232	*F*_1,34_ = 0.68; *p* = 0.417	*F*_1,35_ = 1.42; *p* = 0.241	***F***_**1,62**_ **=** **8.51**; ***p*** = **0.005**
	Year	*F*_1,30_ = 0.79; *p* = 0.380	*F*_1,34_ = 0.34; *p* = 0.564	–	–
3+	Site	*F*_1,28_ = 0.06; *p* = 0.803	*F*_1,32_ = 3.42; *p* = 0.074	*F*_1,24_ = 0.34; *p* = 0.565	*F*_1,51_ = 0.50; *p* = 0.483
	Year	*F*_1,28_ = 0.27; *p* = 0.610	***F***_**1,32**_ = **8.63**; ***p*** = **0.006**	–	–
4+	Site	*F*_1,18_ = 3.75; *p* = 0.071	*F*_1,23_ = 0.42; *p* = 0.524	*F*_1,15_ = 0.55; *p* = 0.470	*F*_1,41_ = 0.53; *p* = 0.473
	Year	*F*_1,18_ = 2.95; *p* = 0.582	***F***_**1,23**_ = **5.05**; ***p*** = **0.035**	–	–
5+	Site	***F***_**1,17**_ = **13.96**; ***p*** = **0.002**	*F*_1,9_ = 0.13; *p* = 0.725	*F*_1,13_ = 0.54; *p* = 0.476	***F***_**1,35**_ = **6.31**; ***p*** = **0.017**
	Year	*F*_1,17_ = 1.32; *p* = 0.269	–	–	–
6+	Site	***F***_**1,16**_ = **4.76**; ***p*** = **0.047**	***F***_**1,6**_ = **18.37**; ***p*** = **0.005**	–	*F*_1,33_ = 1.49; *p* = 0.230
	Year	*F*_1,16_ = 1.66; *p* = 0.218	–	–	–
7+	Site	*F*_1,16_ = 0.13; *p* = 0.722	–	–	–
	Year	*F*_1,16_ = 0.17; *p* = 0.691	–	–	–
8+	Site	*F*_1,10_ = 1.42; *p* = 0.264	–	–	–
	Year	*F*_1,10_ = 0.11; *p* = 0.751	–	–	–

Significant effects are in bold

**Table 3:** The effects of chub age (*A*), site (*S*; invaded versus uninvaded) and year (*Y*; 2008 vs 2011; Rother and Chad Brook only) on chub fork length (*L*), and mass (*M*)

Model	River	Term	a (±SE)	Test statistic	*p*	d.f.	*R* ^2^ *adj*
*L* = a*A* + a*S* + a*Y*	Rother	Full model	–	32.43	<0.001	31	75.3 %
		*A*	31.06 (±3.68)	8.44	<0.001	–	–
		*S*	8.67 (±20.48)	0.42	0.675	–	–
		*Y*	−8.80 (±8.40)	−1.05	0.303	–	–
*L* = a*A* + a*S* + a*Y*	Chad Brook	Full model	–	55.80	<0.001	35	82.5 %
		*A*	28.63 (±3.16)	9.06	<0.001	–	–
		*S*	−39.60 (±10.90)	−3.63	<0.001	–	–
		*Y*	6.76 (±4.38)	4.38	0.133	–	–
*L* = a*A* + a*S*	Cherwell	Full model	–	507.30	<0.001	203	83.3 %
		*A*	32.57 (±1.02)	31.83	<0.001	–	–
		*S*	−0.71 (±3.19)	0.22	0.824	–	–
*L* = a*A* + a*S*	Evenlode	Full model	–	975.90	<0.001	299	86.7 %
		*A*	36.48 (±1.01)	36.09	<0.001	–	–
		*S*	−41.16 (±3.32)	−12.40	<0.001	–	–
*M* = a*A* + a*S* + a*Y*	Rother	Full model	–	23.22	<0.001	31	68.4 %
		*A*	135.43 (±19.46)	6.96	<0.001	–	–
		*S*	158.98 (±108.27)	1.47	0.153	–	–
		*Y*	−46.39 (±44.39)	−1.05	0.305	–	–
*M* = a*A* + a*S* + a*Y*	Chad Brook	Full model	–	55.60	<0.001	35	82.4 %
		*A*	96.35 (±11.49)	8.38	<0.001	–	–
		*S*	−171.11 (±39.64)	−4.32	<0.001	–	–
		*Y*	−4.08 (±15.94)	−0.26	0.800	–	–
*N* = a*L* + a*S* + a*Y*	Rother	Full model	–	3.73	0.023	31	20.9 %
		*L*	0.002 (±0.002)	0.72	0.479	–	–
		*S*	−0.457 (±0.354)	−1.29	0.208	–	–
		*Y*	0.138 (±0.188)	0.73	0.469	–	–
*N* = a*L* + a*S* + a*Y*	Chad Brook	Full model	–	3.60	0.024	35	18.2 %
		*L*	0.005 (±0.003)	1.60	0.119	–	–
		*S*	0.634 (±0.298)	2.13	0.041	–	–
		*Y*	−0.199 (±0.136)	−1.47	0.151	–	–

We also tested the effects of chub fork length, site, and year on chub baseline corrected δ^15^N (*N*) for the Rother and Chad Brook. Test statistics were *F* and *t* for full model and individual terms respectively

There was a linear relationship between chub scale and muscle δ^13^C (*F*_1,13_ = 40.17, *p* < 0.001), and between scale and muscle δ^15^N from Calverton fish farm (*F*_1,13_ = 60.51, *p* < 0.001). Muscle tissue was ^13^C-depleted (mean ± SD: −2.2 ± 0.5 ‰) and ^15^N-enriched (0.8 ± 0.3 ‰) relative to scale tissue and the corresponding regression equations shown (Supplementary Information) were used for converting scale isotope ratios for further comparisons.

**Fig. 3:**
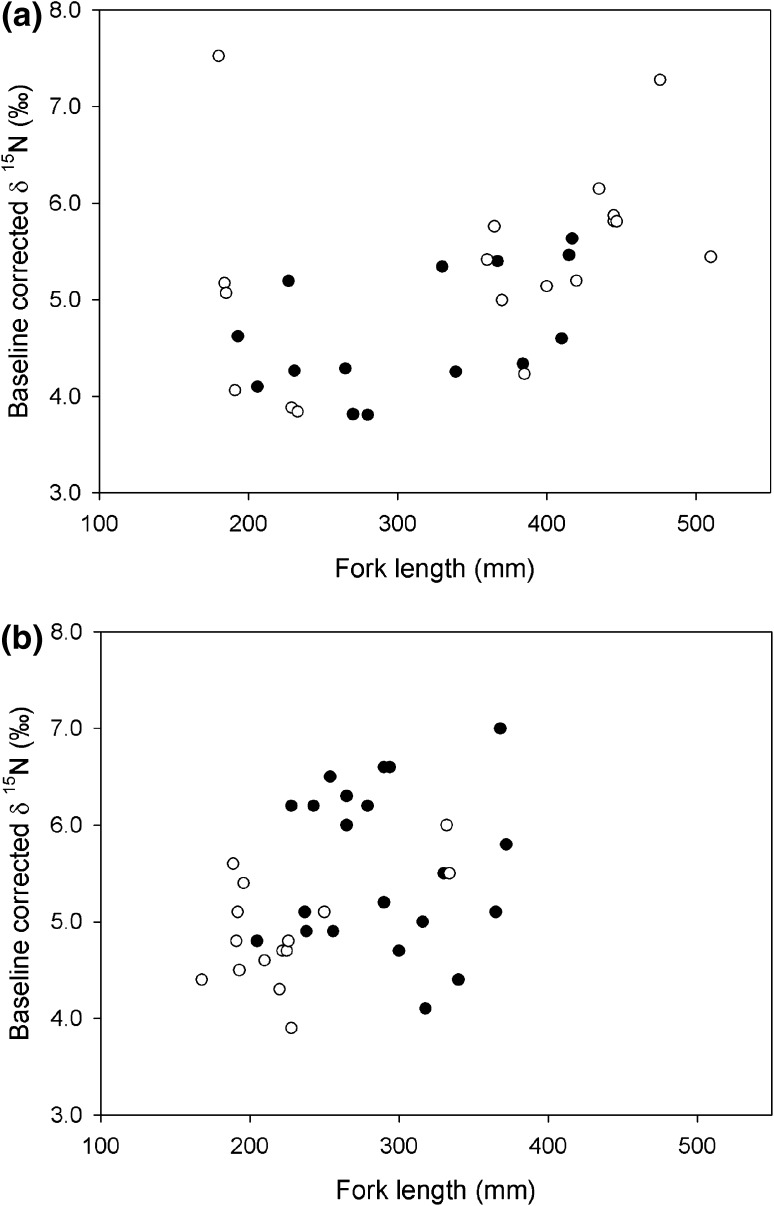
Baseline corrected chub δ^15^N as a function of fork length for uninvaded (*solid circles*) and invaded (*open circles*) sites on **a** the Rother and **b** Chad Brook

**Fig. 4:**
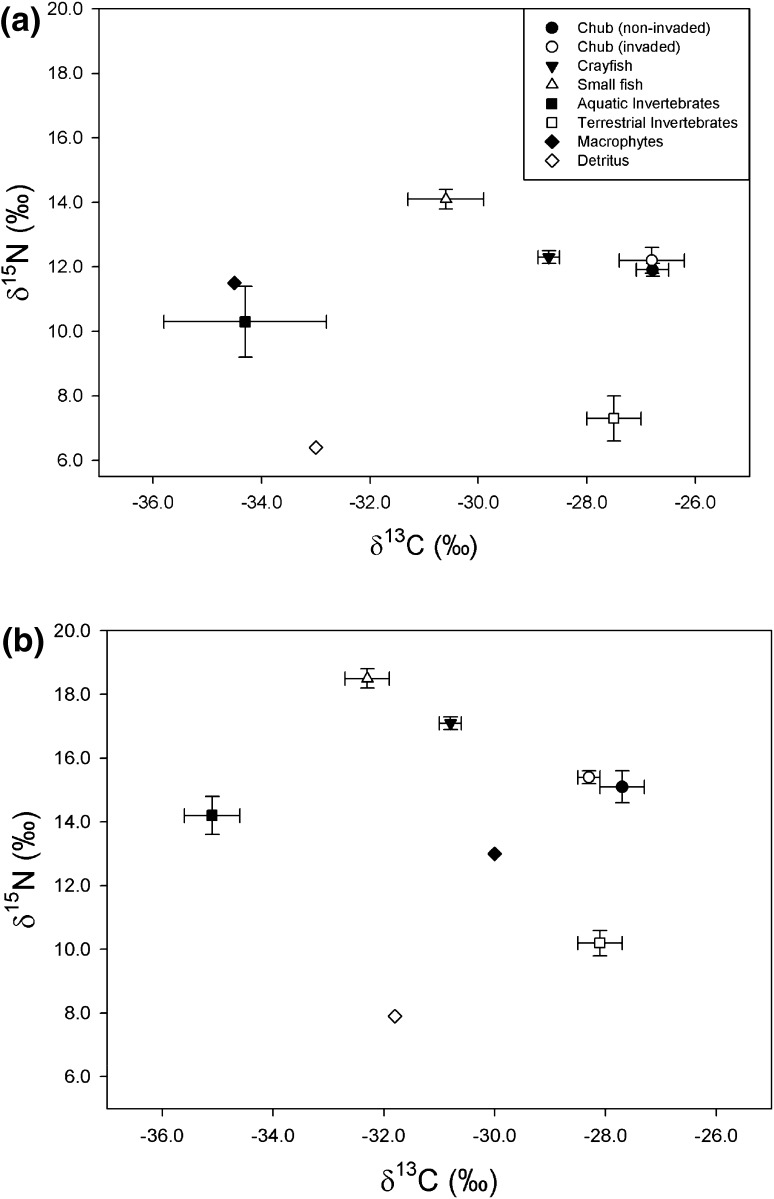
Isotope bi-plots indicating the mean (±standard error) for chub, crayfish, and the putative prey of both species, for **a** the Rother and **b** Chad Brook. For the Rother small fish were 1+ cyprinids, *Phoxinus phoxinus*, *Cottus gobio*, and *Barbatula barbatula*, aquatic invertebrates were Trichoptera, Gammarids, and Ephemeroptera, and terrestrial invertebrates were Formicidae, Arachnidae, Hemiptera, Diptera, and Coleoptera. For Chad Brook small fish were *Phoxinus phoxinus*, *Cottus gobio*, *Barbatula barbatula*, and *Gasterosteus aculeatus*, aquatic invertebrates were Gammarids, *Calopteryx* sp., Heteroptera, Limnaea, and Trichoptera, and terrestrial invertebrates Formicidae, Arachnidae, Diptera, Coleoptera, and Gastropoda

We found no evidence that chub muscle baseline-corrected δ^15^N was related to fork length or sampling year at either the Rother or Chad Brook (Fig. 3; Table 3). However, chub baseline-corrected δ^15^N was higher at the invaded site on Chad Brook, but no differences were detected for the Rother (Fig. 3; Table 3). The relative trophic positions of chub and crayfish, as inferred from isotopic bi-plots (Fig. 4), indicate that both species fed on multiple food sources. Large chub from the invaded Rother site had a mean (±95 % CI) trophic height (measured as δ^15^N) of 5.5 ± 0.9 ‰, compared with 4.7 ± 0.7 at the uninvaded site (Table 4). Similarly, small chub at the invaded site had a trophic height of 5.1 ± 1.5 compared with 4.5 ± 0.5 at the uninvaded site. The mean trophic heights of large and small chub from the invaded site were 1.1 and 0.8 ‰ higher, respectively, than that of crayfish. However, in Chad Brook both large and small chub were estimated to have similar trophic heights in invaded and uninvaded sites, with crayfish trophic height similar to those of chub (Table 4).

**Table 4:** The trophic position of chub and crayfish, as measured by the perpendicular distance from a linear regression fitted to six basal resources (sloping isotope baseline) to the consumer δ^15^N value

River	Group	Baseline corrected δ^15^N (‰)	
		Mean	±95 % CI
Rother			
Uninvaded	Small chub	4.54	0.48
	Large chub	4.69	0.44
Invaded	Small chub	5.14	1.27
	Large chub	5.46	0.47
	Crayfish	4.35	0.30
Chad Brook			
Uninvaded	Small chub	5.53	1.36
	Large chub	5.58	0.38
Invaded	Small chub	4.74	0.26
	Large chub	5.52	0.48
	Crayfish	5.48	0.30

**Fig. 5:**
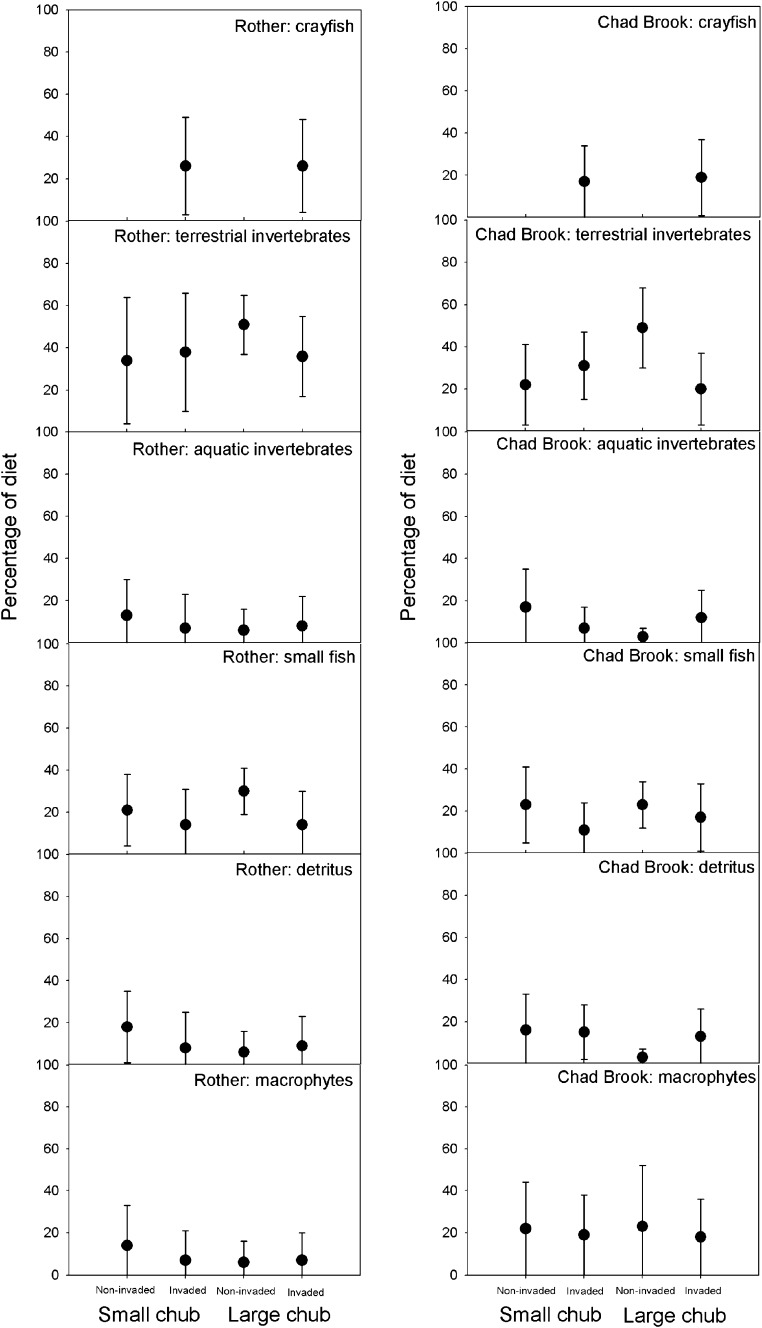
The mean (±95 % CI) percentage of small and large chub diets comprised by each food source at uninvaded and invaded sites, as indicated by the SIAR mixing model. Normal distributions of isotope data were confirmed by visual inspection of the data

SIAR model ouputs indicated that terrestrial invertebrates were the most important prey resource for chub, comprising up to 50 % of chub diet (Fig. 5; Supplementary Information). In contrast, aquatic invertebrates (other than crayfish) constituted <20 % of chub diet for all sites on both rivers. Furthermore, small chub relied even less on aquatic invertebrates at invaded sites, declining from 13 to 7 % in the Rother and from 17 to 7 % in Chad Brook. At invaded sites signal crayfish were estimated to make a mean contribution of up to 26 and 19 % of chub diet in the Rother and Chad Brook, respectively. Similar dietary use of crayfish was found for both size classes of chub. For both rivers the contribution of small fish to chub diet was found to be reduced at the invaded sites. Crayfish exhibited a high degree of omnivory in both rivers, with modelled dietary contributions showing wide ranges for all potential food sources (Supplementary Information). Crayfish in Chad Brook showed greater consumption of specific taxa, with aquatic invertebrates and small fish making mean dietary contributions of 42 and 20 %, respectively. Cannibalism among signal crayfish was estimated to make a mean contribution of 12 % to crayfish diet in both rivers.

## Discussion

Many studies have reported on the negative impacts of invasive species, but there have been relatively few on how the influence of an invader may be beneficial to a recipient system (Caldow et al. 2007; Letnic et al. 2009; Tablado et al. 2010). Our study illustrates that there can be both beneficial and detrimental consequences of invaders on a native species, with the life stage of the native species influencing whether the impact was positive or negative. The growth rates of young individuals of a native fish were reduced when in sympatry with a non-native crustacean, yet some older fish exhibited increased growth rates in the presence of the invader. Our isotope mixing models suggests that crayfish were incorporated as an additional dietary component at invaded sites. Therefore, the influence of the invasive crayfish may be perceived as both negative and positive to chub.

At all sites where chub existed in sympatry with signal crayfish, the 0+ fish exhibited lower growth rates, and this was maintained in the Evenlode until fish were aged 2+. Signal crayfish can prey directly upon small fish (Guan and Wiles 1997) and consequently small fish may spend more time engaged in predator-avoidance, limiting foraging opportunities (Light 2005) and reducing growth rates in chub (Allouche and Gaudin 2001). Whilst our results were correlative, a consistent pattern of reduced juvenile chub growth was detected in our four datasets: a space-for-time approach in Evenlode and Cherwell, and a before-after invasion approach in Rother and Chad Brook. Chub growth rates will likely have been further influenced by additional, unmeasured variables, as evidenced by the observed inter-annual differences in chub growth rates in 3 of 13 comparisons. Such inter-annual differences may reflect between-year variation in environmental conditions such as water temperature and flow speed, which are known to influence the growth rates of cyprinid fishes (Cragg-Hine and Jones 1969). Such variables could have interacted with crayfish presence to modulate the effects of crayfish on chub, for example by increasing crayfish numbers or activity (Olsson et al. 2009b). Furthermore, changes in crayfish densities could have affected chub growth rates, as crayfish impacts on native species are typically density-dependent (e.g. Flint and Goldman 1975). Whilst our study did not account for these additional factors, we were still able to detect effects of crayfish invasion on chub growth rates.

Older chub were generally found to exhibit higher growth rates at invaded sites in three of the four rivers studied. The age at which chub from the invaded sites achieved greater growth rates than those of chub from uninvaded sites varied from 5+ to 6+. Increased chub growth rates associated with the presence of signal crayfish may indicate greater predation on crayfish by larger chub, a plausible inference considering the ^15^N- and ^13^C-enrichment of larger chub. Thus the outputs from the mixing models were consistent with the pattern expected of a gape-limited predator of crayfish. Although Evenlode chub aged 6+ exhibited slightly higher growth rates in the post-invasion period, the difference was not significant. Overall, the data upheld our first hypothesis, that juvenile chub growth rates would be lower when sympatric with signal crayfish, and older, larger chub would show the opposite trend.

This study provides evidence that signal crayfish may alter the size structure of chub populations. Chub length-at-age was reduced at two of the four invaded sites tested; methodology did not appear to influence our results, as reduced length-at-age was detected for sites at which before-after invasion site (Evenlode) and space-for-time (Chad Brook) approaches were used. Furthermore, mass-at-age was reduced at one of the two invaded sites tested, whereas no increases either length-at-age or mass-at-age in response to invasion were detected, probably due to decreased growth rates of young chub in the presence of signal crayfish. Lower 0+ growth has been reported to result in smaller annual growth increments across the lifetime of individual chub (Bolland et al. 2007). In the Rother and Cherwell, older (≥5+) chub from the invaded sites were found to attain greater length-at-age than conspecifics at uninvaded sites, despite younger (≤3+) chub from the same invaded sites exhibiting lower length-at-age values. Based on our complementary stable isotope data, we propose that greater length-at-age in some older chub was the result of consuming invasive crayfish. Our results concur with previous findings that predators can achieve higher post-invasion growth rates and ultimate body size either by direct predation of the invasive species or by indirect effects (King et al. 2006). The changes in chub size-at-age have implications for food web structure and the abundances of prey items as energy requirement and prey availability (due to gape-limitation) are related strongly to fish body size (Wieser 1991).

Our second hypothesis, that chub diet would be altered in the presence of crayfish, is confirmed not only through the incorporation of the invasive crayfish into the diet of chub, but also by a reduction in reliance upon aquatic invertebrates by small chub, and reduced reliance on small fish by chub of both size classes, at the invaded sites. A reduction in the contribution of one food source must lead to compensation through greater dependence on another. Hellawell (1971b) reported that where larger chub exhibit reduced consumption of terrestrial invertebrates, there was increased consumption of fish, frogs and native crayfish. It is therefore likely that an invasive crayfish would be increasingly exploited in the same manner. Furthermore, considering the documented negative effects of crayfish on aquatic invertebrates and macrophytes, it seems less likely that these groups should be more heavily relied upon by the chub of the invaded sites. Indeed, the reduced contribution of aquatic invertebrates (other than crayfish) to chub diet at invaded sites is consistent with previous research that found invasive signal crayfish reduced the total numbers of aquatic invertebrate by 60 % (Crawford et al. 2006). We found some evidence that the potential incorporation of crayfish into the diets of larger chub resulted in elevated baseline corrected δ^15^N values at Chad Brook, but not at the Rother. Chub at invaded sites incorporated high δ^15^N crayfish into their diet, which likely raised large chub δ^15^N at invaded sites. SIAR output indicated greater consumption of terrestrial invertebrates by chub compared to crayfish. Morphology and behaviour limits crayfish primarily to benthic foraging (Guan and Wiles 1998) and therefore precludes access to surface drifting prey. However, once terrestrial invertebrates sink they become available to crayfish. In contrast, chub utilise the entire water column from benthos to surface when foraging (Hellawell 1971b).

The combined growth data and stable isotope evidence does not support our third hypothesis; that changes in growth rate and dietary shifting would be more pronounced in younger chub. In contrast, the increase in growth rates of older chub at the Rother and Chad Brook were greater in magnitude than those differences between the 0+ fish. However, as isotope data were only acquired for ≥3+ chub the impact of crayfish invasion on the diet and trophic position of ≤2+ individuals, which exhibited lower growth rates in sympatry with signal crayfish, remain unknown.

We have shown that crayfish invasion can have both positive and negative impacts on the diet and growth of a native fish, using signal crayfish and chub as exemplars. We found some evidence that older chub benefit from the inclusion of crayfish in their diet and can achieve higher growth rates. Younger chub suffer decreased growth rates, probably due to increased predator-avoidance and reduced prey availability. Potentially, an individual chub may experience both negative and positive impacts of invasion as it progresses through different life stages. Whether crayfish invasion can be considered beneficial or detrimental to the native fish population as a whole will depend, at least in part, on whether the lower juvenile growth rates translate into lower recruitment and thus reduced population size. Further longer-term studies of the population level consequences of crayfish invasion are required. Several studies have found lower populations in the presence of signal crayfish (Guan and Wiles 1997; Peay et al. 2009), whereas others have not (Stenroth and Nyström 2003; Degerman et al. 2007); comprehensive studies of fish populations pre- to post-invasion and assessed relative to the ‘natural state’ (i.e. with the presence of native crayfish) are required to address this question.

## Electronic supplementary material

Below is the link to the electronic supplementary material.

Supplementary material 1 (DOCX 54 kb)
