# Supplementary material for: Invasive crayfish impacts on native fish diet and growth vary with fish life stage
Source: Aquat Sci. 2016 Apr 22;79(1):113–25. doi: 10.1007/s00027-016-0483-2 (PMC7115035; doi:10.1007/s00027-016-0483-2)
Supplement: Supplementary file 1 — Supplementary material 1 (DOCX 54 kb) [file 27_2016_483_MOESM1_ESM.docx]

**Invasive crayfish impacts on native fish diet and growth vary with fish life stage**

Kevin A. Wood, Richard B. Hayes, Judy England and Jonathan Grey

**Supplementary Information**

Figure S1:

**Fig. S1:** Relationships between chub scale and muscle tissue isotope values (δ^13^C and δ^15^N) from Calverton Fish Farm. Regression lines are y = 0.87x - 4.63 for δ^13^C (R^2^ = 75.6 %), and y = 0.74x + 3.35 for δ^15^N (R^2^ = 82.3 %).

|  | **Crayfish** | | **Terrestrial invertebrates** | | **Aquatic invertebrates** | | **Small fish** | | **Detritus** | | **Macrophytes** | | **Chub** | |
| --- | --- | --- | --- | --- | --- | --- | --- | --- | --- | --- | --- | --- | --- | --- |
|  | Mean | 95 % CI | Mean | 95 % CI | Mean | 95 % CI | Mean | 95 % CI | Mean | 95 % CI | Mean | 95 % CI | Mean | 95 % CI |
| **Uninvaded Rother** |  |  |  |  |  |  |  |  |  |  |  |  |  |  |
| Small chub | n.a. | n.a. | 34 | 8 – 67 | 13 | 0 – 30 | 21 | 3 – 37 | 18 | 0 – 35 | 14 | 0 – 33 | n.a. | n.a. |
| Large chub | n.a. | n.a. | 51 | 38 – 63 | 6 | 0 – 16 | 30 | 19 – 41 | 6 | 0 – 16 | 6 | 0 – 16 | n.a. | n.a. |
| **Invaded Rother** |  |  |  |  |  |  |  |  |  |  |  |  |  |  |
| Small chub | 26 | 1 – 49 | 38 | 10 – 66 | 7 | 0 – 23 | 14 | 0 – 31 | 8 | 0 – 25 | 7 | 0 – 21 | n.a. | n.a. |
| Large chub | 26 | 3 – 48 | 36 | 16 – 54 | 8 | 0 – 22 | 14 | 0 – 30 | 9 | 0 – 23 | 7 | 0 – 20 | n.a. | n.a. |
| Crayfish | 12 | 0 – 26 | 19 | 4 – 33 | 15 | 13 – 26 | 10 | 0 – 24 | 19 | 4 – 33 | 13 | 0 – 26 | 11 | 0 – 23 |
| **Uninvaded Chad Brook** |  |  |  |  |  |  |  |  |  |  |  |  |  |  |
| Small chub | n.a. | n.a. | 22 | 3 – 41 | 17 | 0 – 36 | 23 | 3 – 39 | 16 | 0 – 33 | 22 | 0 – 41 | n.a. | n.a. |
| Large chub | n.a. | n.a. | 49 | 28 – 68 | 3 | 0 – 7 | 23 | 10 – 34 | 3 | 0 – 7 | 23 | 0 – 52 | n.a. | n.a. |
| **Invaded Chad Brook** |  |  |  |  |  |  |  |  |  |  |  |  |  |  |
| Small chub | 17 | 0 – 34 | 31 | 15 – 47 | 7 | 0 – 17 | 11 | 0 – 24 | 15 | 1 – 26 | 19 | 0 – 38 | n.a. | n.a. |
| Large chub | 19 | 1 – 36 | 20 | 2 – 35 | 12 | 0 – 25 | 17 | 1 – 32 | 13 | 0 – 26 | 18 | 0 – 34 | n.a. | n.a. |
| Crayfish | 12 | 0 – 28 | 5 | 0 – 12 | 42 | 27 – 57 | 20 | 1 – 37 | 7 | 0 – 16 | 8 | 0 – 20 | 7 | 0 – 17 |

**Table S1:** SIAR output for chub and crayfish of the rivers Rother and Chad Brook, indicating the relative contribution of each food source to chub and crayfish diets. For each potential food source an estimated mean (and 95 % confidence interval) contribution is given, expressed as a percentage of the diet. The term n.a. indicates a food source was unavailable to chub.
